# Distal interphalangeal joint arthrodesis with nonaxial multiple small screws: a biomechanical analysis with axial headless compression screw and clinical result of 15 consecutive cases

**DOI:** 10.1186/s12891-022-05473-9

**Published:** 2022-05-27

**Authors:** Seung Hun Woo, Sang Ho Kwak, Hyo Seok Jang, Dong Hee Kim, Jang Hyeon Seo, Sang Hyun Lee

**Affiliations:** 1 Department of Orthopaedic Surgery, Pusan National University Yangsan Hospital, Yangsan, Republic of Korea; 2 Department of Orthopaedic Surgery, Inje University Haeundae Paik Hospital, Busan, Republic of Korea; 3 Department of Orthopaedic Surgery, Samsung Changwon Hospital, Sungkyunkwan University School of Medicine, 58, Paryong-ro, Masanhoewon-gu, Changwon-si, 513-53 Republic of Korea; 4 Jeil Medical Corporation, Digital-ro 34, Seoul, Republic of Korea; 5 Department of Orthopaedic Surgery, Medical Research Institute, Pusan National University Hospital, Pusan National University School of Medicine, 179, Gudeok-ro, Seo-gu, Pusan, Korea, 602-739 Busan, Republic of Korea

**Keywords:** Osteoarthritis, Arthrodesis, Finger joint, Small screw, Headless compression screw, Biomechanical

## Abstract

**Background:**

The axial headless compression screw (AHCS) technique is a widely used method for distal interphalangeal joint (DIPJ) and thumb IPJ arthrodesis. However, it might not be suitable for cases over 10° flexion of fusion angle and extremely small-sized phalanx. Here, the authors describe the nonaxial multiple small screws (NMSS) technique, compare the mechanical strength of the NMSS technique with the AHCS technique, and suggest clinical outcomes of the NMSS technique.

**Methods:**

DIPJ and thumb IPJ arthrodesis models were simulated in the 4th generation composite bone hand. Fixation with three 1.5 mm cortical screws (NMSS) or one HCS (AHCS) was performed in each pair of the phalanx. The bending stiffness and load to failure were tested in 10 pairs of each specimen, and the torsional stiffness and torque to failure were tested in seven pairs of each specimen. Moreover, 15 consecutive clinical DIPJ and thumb IPJ arthrodesis cases were reviewed retrospectively.

**Results:**

The NMSS specimens showed significantly higher bending load to failure, torsional stiffness, and torque to failure than the AHCS specimens. All 15 arthrodesis cases were united without severe complications. The mean fusion angle was 16.3° for the nine cases of the flexed target position.

**Conclusions:**

The NMSS technique showed biomechanical stability comparable to that of the AHCS technique in DIPJ and thumb IPJ arthrodesis. Thus, the NMSS technique could be used as a feasible option in DIPJ and thumb IPJ arthrodesis, especially when a small finger is indicated and a significant flexion angle is required.

**Supplementary Information:**

The online version contains supplementary material available at 10.1186/s12891-022-05473-9.

## Background

Arthrodesis is a good surgical option for painful arthritis of the distal interphalangeal joint (DIPJ) and thumb interphalangeal joint (IPJ) of the hand. Many surgical techniques for arthrodesis have been used, including Kirschner wires (K-wires), cerclage wires, tension band wires, cortical screw insertion, and headless compression screw (HCS) insertion [1–3]. Among them, the axial HCS (AHCS) technique has been reported to be widely used and has proven its biomechanical superiority over other techniques [4, 5], with lower infection and nonunion rates [1]. Besides, the AHCS technique could provide compression of the fusion surface [6]. However, axial screw insertion along the medullary canal is not suitable if a fusion angle of more than 10° flexion is needed for improved grip function and power [7–9], and HCS cannot be used in extremely small-sized fingers due to the risk of size-related complications [1, 10, 11]. Thus, surgical options other than the AHCS technique are needed for cases requiring a significantly large fusion angle and cases with small fingers.

Except for the K-wire, cerclage wire, or tension band wires which have been proven to be disadvantageous biomechanically, nonaxial cortical screw or HCS insertion have been used in the IPJ arthrodesis [12, 13], and nonaxial multiple HCSs were used for proximal interphalangeal joint (PIPJ) arthrodesis [14]. Under these theoretical backgrounds, we have previously used three or more small cortical screws for DIPJ and thumb IPJ arthrodesis (nonaxial multiple small screws; NMSS). As far as we know, this method has not been described yet and the biomechanical stability and clinical outcome of this technique have not been reported. Thus, the purpose of the present study was 1) to describe the NMSS technique for DIPJ and thumb IPJ arthrodesis and compare the mechanical strength of the NMSS technique with the AHCS technique in a biomechanical composite bone model and 2) to suggest the clinical outcome of DIPJ and thumb IPJ arthrodesis cases using the NMSS technique.

## Material and methods

### Biomechanical study

#### Sample size estimation

The null hypothesis of this study was that the NMSS technique is biomechanically non-inferior to the AHCS technique in terms of bending and torsional stability. Differences in bending stability between PIPJ arthrodesis techniques have been reported [15]. From these results, the non-inferiority margin (δ) and standard deviation (SD) for biomechanical variables (the load to failure for bending stability) could be determined [15]. Assuming paired groups, the required sample size of load to failure for bending stability was 8 with a power of 80% and a level of significance of 0.05 (δ = 8, SD = 8). The required sample size for torsional rigidity of DIPJ arthrodesis techniques was more than 4 (δ = 45,410, SD = 24,310) [4].

#### Preparing samples

**Fig. 1:**
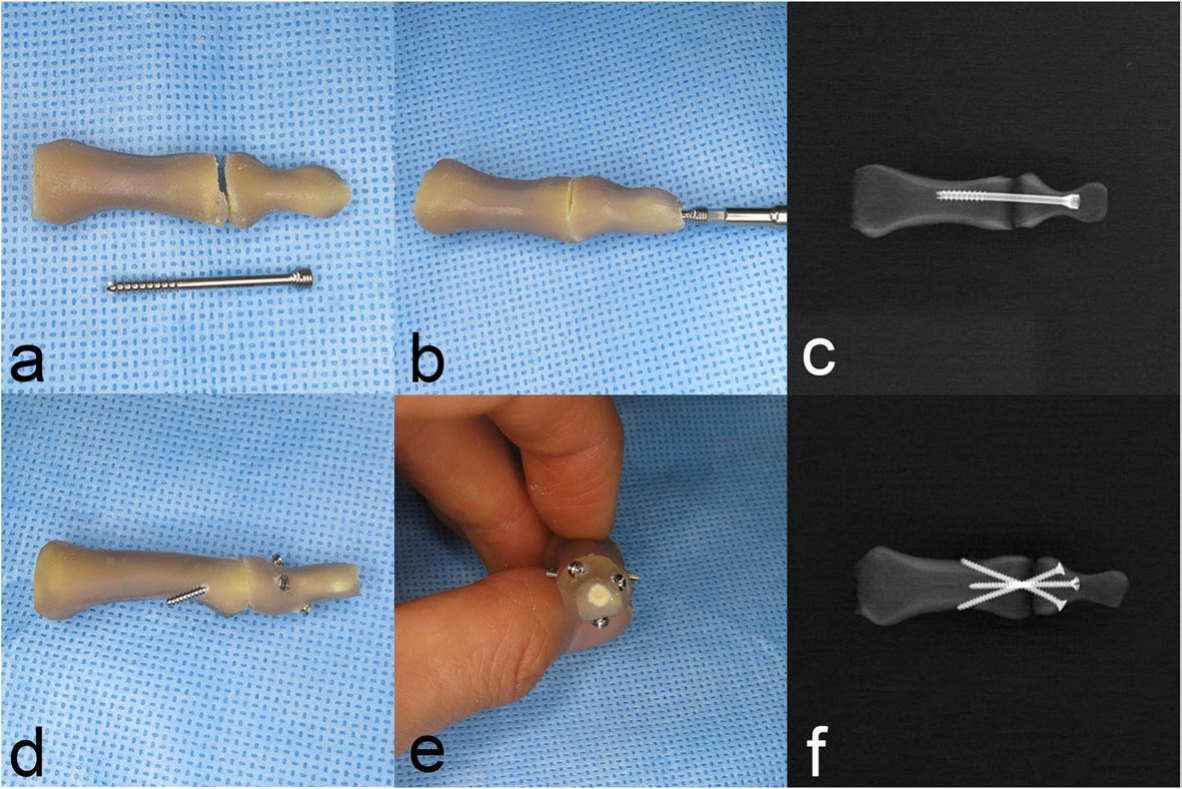
**a** ~ **c** A distal interphalangeal arthrodesis specimen with axial headless compression screw (AHCS) and a posteroanterior radiograph of the AHCS specimen. A headless compression screw of 28 mm length was intramedullary inserted; **d** ~ **f** A distal interphalangeal arthrodesis specimen with nonaxial multiple small screws (NMSS) and a posteroanterior radiograph of NMSS specimen show one screw (1.5 mm cortical) inserted unicortically and two screws were inserted bicortically

Fourth generation composite solid foam core hand (Model sku 3420; Pacific Research Laboratories, Vashon Island, WA, USA) was used as the biomechanical bone test medium [16, 17]. The thumb IPJ and DIPJ of the 2^nd^–5^th^ fingers were selected for the specimen. Each articular surface was trimmed perpendicular to the longitudinal axis. The joint was stabilized using the NMSS or AHCS technique. For the NMSS technique, one 1.5 mm screw (15-HC-020, Jeil Medical Corporation, Seoul, KOR; diameter of 1.5 mm and length of 20 mm) was inserted from the volar cortex of the distal phalanx to the medullary canal of the middle phalanx. Two additional 1.5 mm screws were inserted from the distal phalanx to the middle phalanx in a bicortical cross fixation manner. For the AHCS technique, one 2.3 mm headless compression screw (23-CH-028L, Jeil Medical Corporation, Seoul, Korea; trailing thread diameter of 3.1 mm, leading thread diameter of 2.3 mm, and length of 28 mm) was used to fix the joint (Fig. 1) [4]. The constructs were paired according to the number of fingers (e.g., the 3^rd^ fingers were paired). Ten pairs of constructs were prepared in a cantilever bending test, and seven pairs of constructs were prepared for a torsional stability test.

#### Tests setup

**Fig. 2:**
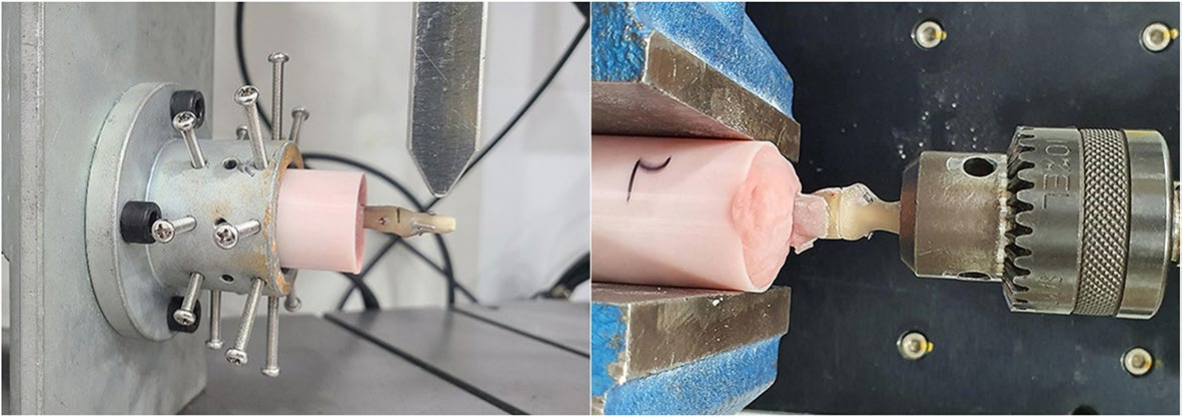
The specimen was secured for the bending stress test and for the torsional stress test

Each specimen was loaded into a universal testing machine (Instron E3000, Norwood, GA, USA). As the specimen was relatively small, the middle phalanx was fixed with a cylindrical resin, and the resin was secured to a steel holder with multiple bolts. The cantilever bending test was divided into a bending fatigue test and a bending load to failure test. The loading unit was placed at the tip of the distal phalanx. For the bending fatigue test, the specimens were loaded from 0 to 20 N at 5 Hz for 3000 cycles, and the data were acquired every 0.1 s. A load of 20 N was used because it compares with the physiologic loads expected with active finger flexion motion [18]. The construct stiffness (N/mm) was measured from the load–displacement ratio. Then, the construct was loaded at a constant rate of 5 mm/min for the bending load to failure test. A load–displacement curve was plotted for each specimen, and the failure load was defined as the first distinctive peak load followed by a sharp drop in the load–displacement curve. For the torsional stability test, the middle phalanx was secured to a steel holder, and the distal phalanx was secured by the testing platform and rotated in a counterclockwise direction at a rate of 0.6 rotations per min. The data were acquired every 0.05 s. The required torque and rotational displacement curves are plotted for each specimen. The construct stiffness was measured using the slope of the linear portion of the graph. A sharp drop in torque was considered to indicate the structural failure of the specimen, and the first peak torque was recorded as torque-to-failure (Fig. 2).

#### Statistical analysis

All statistical analyses were performed using SPSS version 19.0 (IBM Co., Armonk, NY, USA). The Shapiro–Wilk test was used to test for normality and the paired t-test was used to compare parametric continuous variables between matched pairs. Statistical significance was set at *p* < 0.05.

#### Clinical data

The study protocol was approved by the Institutional Review Board (IRB number 05–2021-097); we retrospectively reviewed 15 consecutive cases of 12 patients who underwent DIPJ or thumb IPJ arthrodesis using the NMSS technique between March 2014 and December 2019. Etiologies were either post-traumatic arthritis (n = 3) or osteoarthritis (n = 12), and none were diagnosed with concomitant rheumatoid arthritis [19].

#### Surgical technique and postoperative care

Surgery was performed by one orthopedic surgeon with level 2 (specialist-less experienced faculty) for the NMSS technique [20]. Preoperatively, the patients chose the DIPJ and thumb IPJ angle of arthrodesis in either extension position (0°) for esthetic purpose or flexed position (10–30°) for improved grip function. All surgical procedures were performed under local anesthesia. A finger tourniquet was applied, and an inverted Y-incision was made over the involved joint. The terminal tendon was cut in a z-plasty fashion for an easy repair, and the cartilage was removed until the subchondral bone was exposed. Then, the subchondral bone was carefully trimmed with a small burr to achieve maximal bone contact. Under fluoroscopy, the joint was compressed manually and provisionally fixed with two 1.0 mm K-wires, according to the angle chosen preoperatively. Additional K-wires were introduced to make drill holes for small screws (a 1.0 mm K-wire hole for a 1.2 mm small screw; a 1.2 mm K-wire hole for a 1.5 mm small screw). The ideal drill holes and screw lengths were chosen under fluoroscopy. At least three small screws were used, and additional screws were introduced if there was enough room for the thumb or fingers with large osteophyte that could be fixed. Bicortical screw purchase was advocated, followed by unicortical screw insertion. Between the 1.2 mm and 1.5 mm screw, 1.5 mm screws were considered first; 1.2 mm screws were considered if there was not enough room for 1.5 mm screws. The lag screw technique was not used to decrease iatrogenic comminution of the phalangeal bone [21]. After irrigation the terminal tendon was repaired, and the skin was closed.

The DIPJ or thumb IPJ was protected by an aluminum splint for 6 weeks, and the PIPJ and metacarpophalangeal joint motions were allowed immediately postoperatively. After suture removal at 2 weeks postoperatively, wound desensitization began [22]. At 6 weeks, the splint was changed to a nighttime splint and typing and writing were allowed without protection. Weight-bearing and power-grip motions were allowed after radiographic union was achieved.

#### Data review and outcome assessment

Data regarding laterality, including joint involvement, complications, and additional procedures were reviewed retrospectively. The visual analog scale (VAS) (score range 0–10) for pain in the involved joint and the Quick DASH (Disabilities of the Arm, Shoulder, and Hand) score at the initial and last visits were also reviewed. Finger posteroanterior and lateral radiographs taken at 6 weeks, 3 months, 6 months, and 12 months postoperatively were reviewed. Additionally, the narrowest cortical diameter (outer cortex to outer cortex) of the distal phalanx and middle phalanx were measured on preoperative radiographs [23].

## Results

### Biomechanical outcomes

**Fig. 3:**
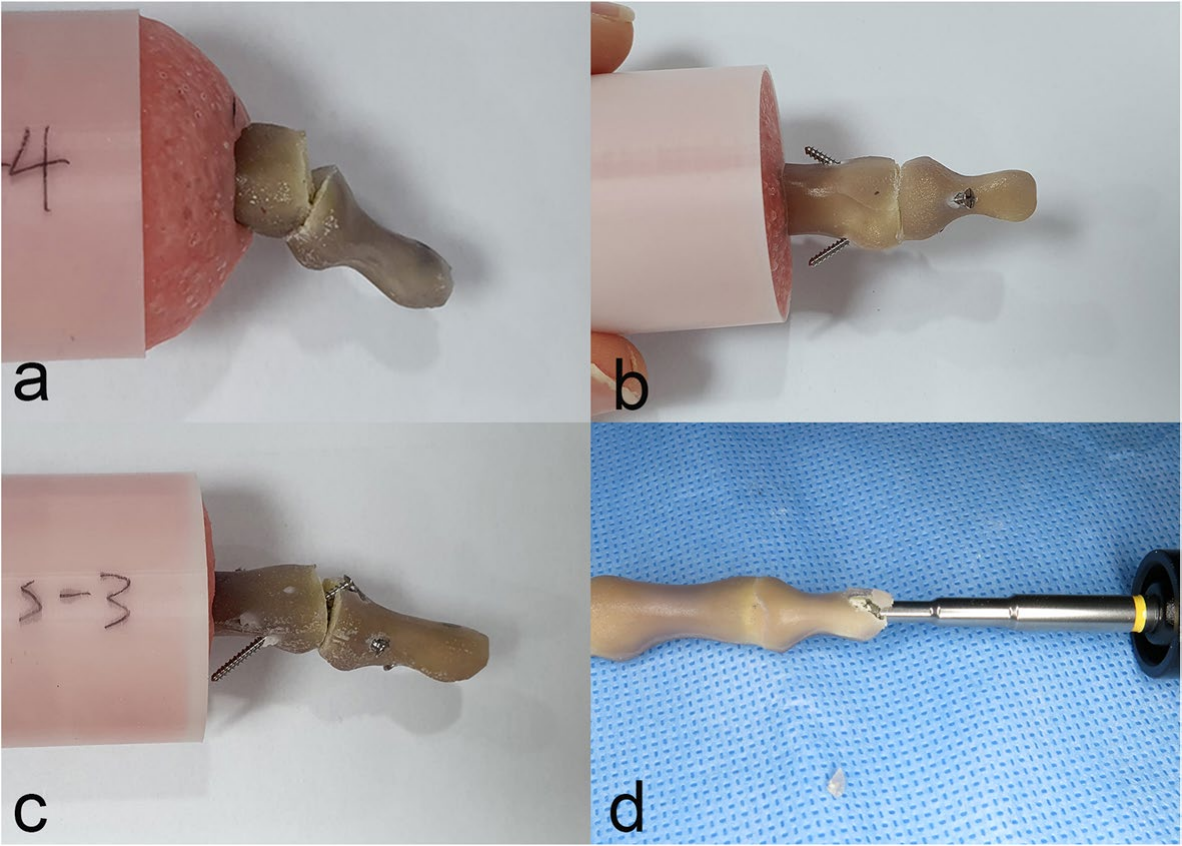
Construct failures in the bending load to failure test. **a** A specimen with the axial headless compression screw technique showed fixation loosening in the medullary canal of the middle phalanx; **b** cortical breakage of the middle phalanx; and **c** that of the distal phalanx in specimens with the nonaxial multiple small screws technique; **d** tip fracture during headless compression screw insertion

**Fig. 4:**
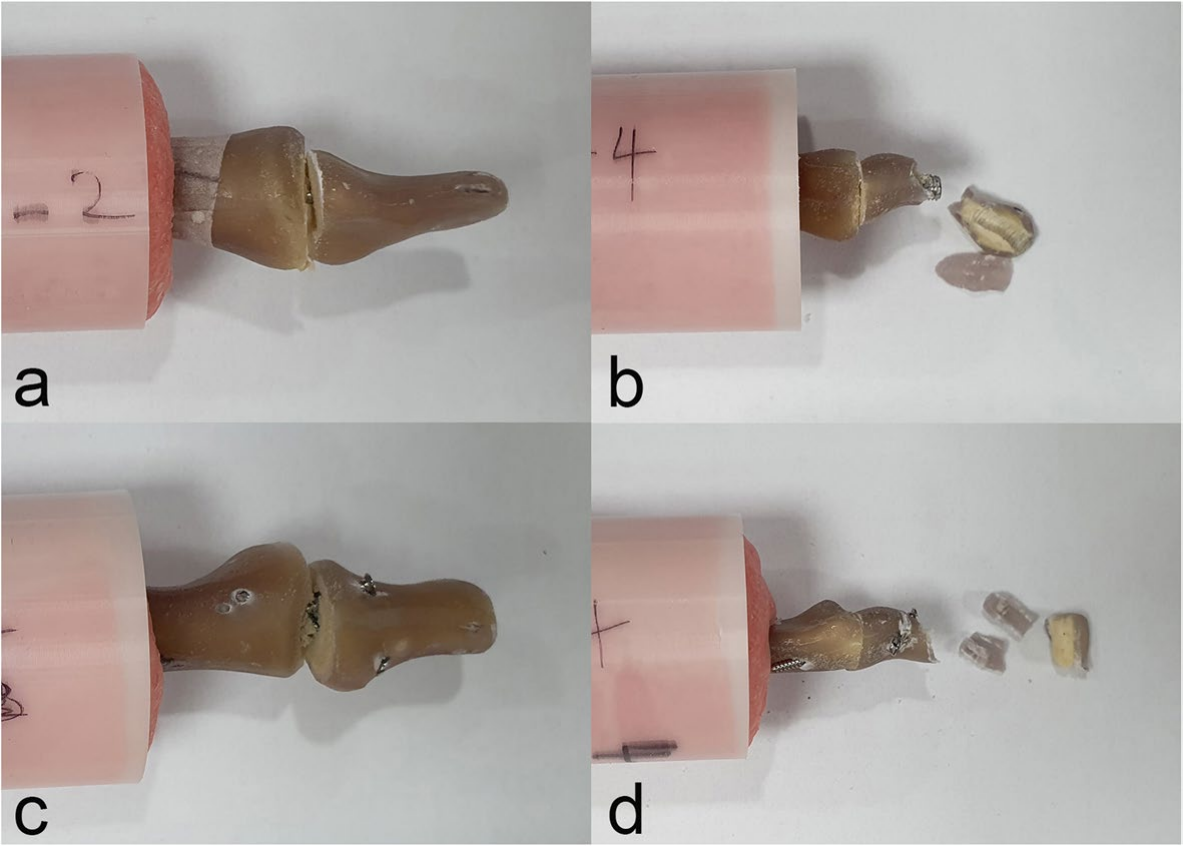
Construct failures in the torque to failure test; **a** Fixation loosening in the medullary canal of the middle phalanx and **b** cortical breakage of the distal phalanx in specimens with the axial headless compression screw technique. **c** Fixation loosening from the cortex of the middle phalanx and **d** cortical breakage of the distal phalanx in specimens with the nonaxial multiple small screws technique

The NMSS specimens showed significantly higher bending load to failure, torsional stiffness, and torque to failure than the AHCS specimens (Table. 1). During the bending fatigue test, no specimen was broken or loosened. During the bending load to failure test, all AHCS specimens showed fixation loosening in the medullary canal of the middle phalanx (*n* = 10), while the NMSS specimens showed cortical breakage of the middle phalanx (*n* = 9) or cortical breakage of the distal phalanx (*n* = 1) (Fig. 3). During the torque-to-failure test, AHCS specimens showed fixation loosening in the medullary canal of the middle phalanx (*n* = 4) or cortical breakage of the distal phalanx (*n* = 3), while the NMSS specimens showed fixation loosening from the cortex of the middle phalanx (*n* = 1) or cortical breakage of the distal phalanx (*n* = 6) (Fig. 4). No implant breakage was observed. Before testing, six AHCS specimens fractured at the distal tip during the attempted insertion and were discarded. No NMSS specimen was fractured during screw fixation.

**Table 1:** Summary of biomechanical result

	AHCS specimens (*n* = 10)	NMSS specimens (*n* = 10)	*p*
Bending stiffness (N/mm) ^a^	23.0 (7.7)	31.6 (17.2)	0.16
Bending load to failure (N) ^a^	72.2 (24.0)	97.4 (26.4)	0.009^†^
	AHCS specimens (*n* = 7)	NMSS specimens (*n* = 7)	
Torsional stiffness (Nm/degrees) ^a^	0.029 (0.013)	0.062 (0.029)	0.026^†^
Torque to failure (Nm) ^a^	0.41 (0.1)	0.99 (0.46)	0.018^†^

*AHCS* axial headless compression screw, *NMSS* nonaxial multiple small screw

^a^ paired t test. Mean ± SD presented

^†^ Statistically significant

### Clinical outcome

**Fig. 5:**
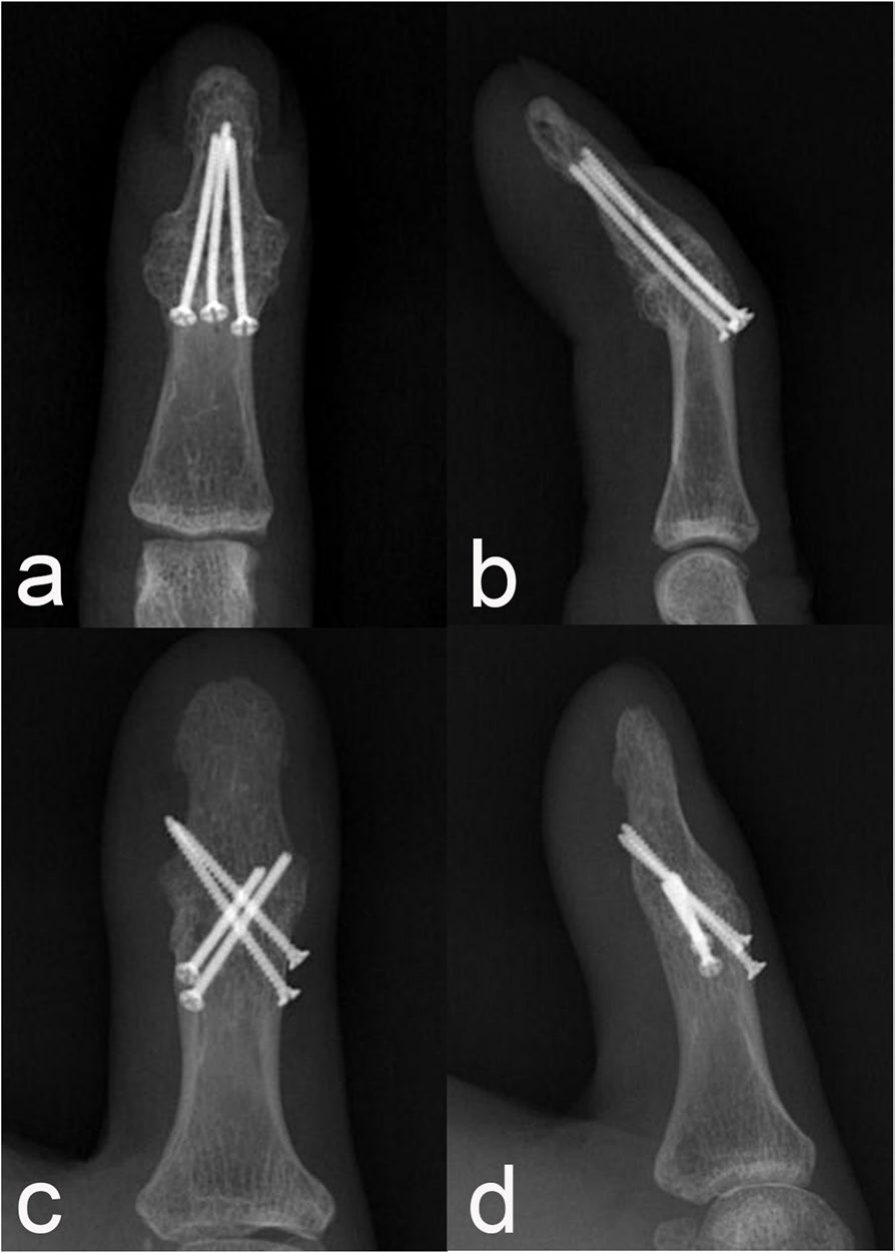
**a**, **b** The 3^rd^ finger of a 63-year-old woman (patient 7). Three 1.5 mm screws were inserted from the middle phalanx to the distal phalanx unicortically with a fusion angle of 35°.; **c**, **d** The thumb of a 41-year-old man (patient 4). Three 1.5 mm screws and one 1.2 mm screw were inserted bicortically with a fusion angle of 13°

**Fig. 6:**
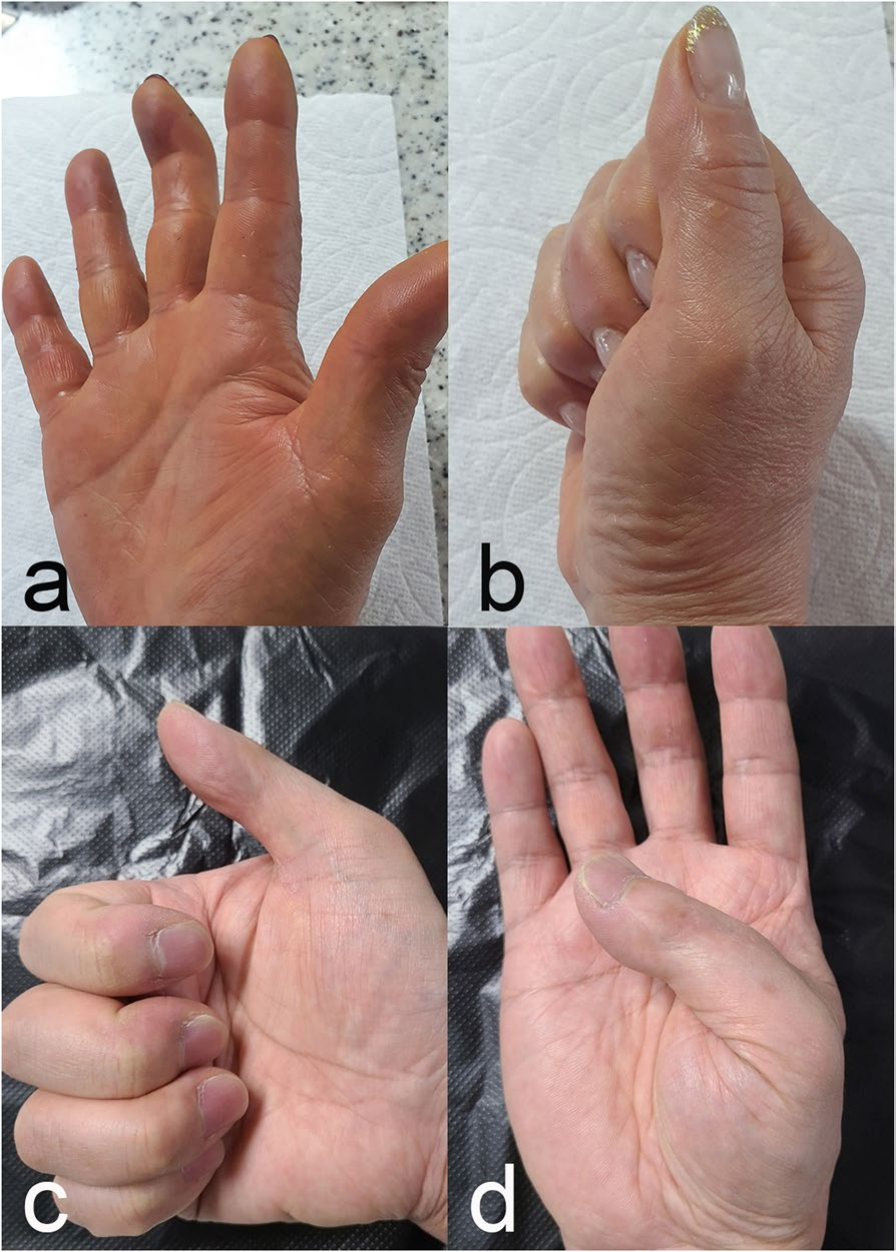
**a**, **b** The 3^rd^ finger of patient 7. The range of motion of the proximal interphalangeal joint and the metetacarpophalangeal joint remained full. **c**, **d** The thumb of patient 4 could touch the metacarpophalangeal joint crease of the ring finger

Radiographic union was observed in all cases at the last follow-up, without metal failure or screw migration. Most cases achieved union within 6 months, except for patient 11, who achieved union at 11 months of follow-up radiograph. The mean fusion angle was 6.3° (range 0–10°) for six cases of extension target position and 16.3° (range 8–35°) for nine cases of flexed target position (Fig. 5, 6). There was no wound dehiscence, infection, nail deformity, or stiffness. However, screws were removed in two patients due to personal preference (patient 4) and hardware irritation (patient 11) (Table. 2). The VAS score for pain at the last visit (mean ± standard deviation; 1.0 ± 0.9) was significantly decreased compared to the VAS score at the initial visit (6.6 ± 1.5, *p* < 0.001). The Quick DASH score at the final visit (20.5 ± 7.8) was significantly improved compared to that at the initial visit (47.7 ± 9.3, *p* < 0.001).

**Table 2:** Clinical results of the patients

Patient	Age(yr)/Sex (M/F)	Laterality (R/L)	Finger location	Narrowest cortical diameter (mm)		Used screws (1.5 mm/1.2 mm)	Duration of operation (min)	DIPJ and thumb IPJ fusion angle (angle°/target position)	Follow up confirming union (Months)	Complication	Quick DASH	
				Distal phalanx (PA/Lat)	Middle phalanx (Proximal for thumb) (PA/Lat)						Preoperative	Postoperative
1	49/F	R	1^st^	6.7/3.7	8.3/4.5	3/1	95	15/flexed	6	-	56.8	18.2
2	65/F	R	3^rd^	5.2/3.8	8.1/5.1	3/0	86	6/extension	6	-	40.9	15.0
3	59/M	R	2^nd^	4.8/3.6	7.2/6.0	3/0	102	7/extension	6	-	59.1	27.3
4	41/M	R	1^st^	9.4/6.7	10.6/7.2	3/1	78	13/flexed	6	Screw removal	45.5	29.5
5	66/M	R	3^rd^	5.9/3.5	8.0/4.8	4/0	88	20/flexed	5	-	47.7	27.3
6	54/F	L	1^st^	6.1/4.3	8.1/4.3	3/1	98	13/flexed	6	-	38.6	9.1
7	63/F	R	3^rd^	3.1/2.7	5.1/3.9	3/0	76	35/flexed	3	-	29.5	15.0
8	59/F	L	1^st^	8.5/5.3	8.8/6.9	3/1	69	15/flexed	6	-	61.4	27.3
		L	4^th^	4.6/4.3	6.3/4.1	2/1	81	12/flexed	3	-		
9	61/F	L	5^th^	3.1/2.5	6.1/3.1	2/1	85	8/extension	5	-	59.1	27.3
10	63/F	R	2^nd^	3.9/3.0	6.3/6.1	3/0	92	10/extension	11	Screw removal	47.7	29.5
11	69/F	L	5^th^	3.1/2.2	5.7/4.2	0/4	82	8/flexed	4	-	47.7	9.1
		R	5^th^	3.4/2.3	6.1/4.3	2/1	95	16/flexed	6	-	45.5	18.2
		R	3^rd^	3.7/2.3	6.3/4.3	3/0	78	0/extension	6	-		
12	62/F	R	3^rd^	4.8/3.1	7.1/3.7	3/0	68	7/extension	6	-	40.9	13.6

*M* male, *F* female, *R* right, *L* left, *PA* posteroanterior, *DIPJ* distal interphalangeal joint, *IPJ* interphalangeal joint, *DASH* Disabilities of the Arm, Shoulder, and Hand

## Discussion

Our results showed that NMSS specimens were not inferior to the AHCS specimens in terms of the tested biomechanical properties, and all DIPJ and thumb IPJ arthrodesis cases using the NMSS technique achieved union without severe clinical complications.

In the bending fatigue test, none of the specimens showed fixation loss within 20 N although the NMSS specimens showed higher stiffness than the AHCS specimens. Thus, we assumed that active range of motion exercise of PIPJ and metacarpophalangeal joint could be initiated before the joint union, in either the AHCS technique or in the NMSS technique [18]. However, the maximal torque in the transverse plane of the DIPJ was reported to range from 0.28 − 1.28 Nm during forceful manipulation of objects (e.g., jar opening) [24]. Considering the mean torques to failure in the current study were 0.41 Nm and 0.99 Nm, we suggest that forceful manipulation of objects should be postponed until the joint union is achieved in both techniques.

During the load to failure test, 14 specimens with AHCS showed loosening in the medullary canal at the middle phalanx, and three showed cortical breakage of the distal phalanx. Since the narrowest cortical diameter was reported to be larger in the middle phalanx than in the distal phalanx, and the leading diameter was smaller than the trailing thread diameter [23], we assumed that the leading diameter might not be large enough to fit the inner cortex of the middle phalanx in some specimens. Moreover, the leading thread is often not placed at the narrowest cortical level if too long or short screws are used. Consequently, the leading thread might be placed in the cancellous bone without sufficient contact with the cortical bone. Therefore, fixation loss might start from the middle phalanx without cortical breakage in most AHCS specimens. Meanwhile, if fixation loss started in the distal phalanx, cortical breakage was always observed. A similar study using Herbert screws with a larger trailing thread diameter (3.9 mm) reported distal phalanx cortical breakage in the load to failure test [4]; thus, we assumed that eccentric reaming and a relatively large trailing thread diameter might cause focal cortical thinning of the distal phalanx and eventually cause cortical breakage. Moreover, the cortex of the distal phalanx of the six AHCSs broke during screw insertion. Thus, surgeons using the AHCS technique should consider appropriate leading thread diameter and screw length to contact the cortical bone at the middle phalanx and should be careful not to cause excessive cortical damage of the distal phalanx, including iatrogenic fracture.

The trailing thread diameter of commercially available HCS was 2.5 mm or greater [10, 23]. However, patients 9 and 11 in our clinical cases had a 2.5 mm or narrower cortical diameter at the distal phalanx, and there was a risk of distal phalanx fracture even if we used the AHCS technique. Alternately, the NMSS technique uses relatively small diameter screws (1.2 mm or 1.5 mm), does not need reaming distal phalanx, and does not purchase dorsal cortex of the distal phalanx. Thus, the NMSS technique might not cause size-related complications reported in the AHCS technique; we did not experience these complications in our cases. Additionally, small screws can be inserted in various directions, such as parallel pinning, crossed pinning, unicortical fixation, bicortical fixation, antegrade insertion, or retrograde insertion. This could allow various DIPJ flexion angles of arthrodesis other than 0° (in our cases, 16.3° for flexed target position), which is important for greater grip strength and dexterity to regain early sports activity [7, 9]. Although the NMSS technique allows various arthrodesis angles, it is technically demanding to acquire full extension position (0°) without axial intramedullary screw insertion. In our cases, the arthrodesis angle was 10° in patient 10 who wanted the extension position. Besides, NMSS technique was more time consuming compared to AHCS technique (unpublished data, Additional File 1). Moreover, hardware under soft tissue might cause skin irritation and delayed infection. Thus, the AHCS technique might be considered first in large-sized phalanges with extension target position, and the NMSS technique might be used in small-sized phalanges or flexed target positions. The prominent advantage of NMSS technique may be that it could be used for arthrodesis of these two difficult cases.

In the clinical setting, there may be several other advantages of the NMSS technique. First, surgeons can choose the number and size of screws for fixation depending on the individual size of the phalanx. Additional screws can be used for further stability in a large-sized phalanx and 1.2 mm screws can be used in a small-sized phalanx. Second, there is more room for errors in the NMSS technique. Usually, 1.0 mm or 1.2 mm K-wires can be used for provisional interphalangeal fixation without bony comminution. Some authors even reported good result with only K-wires in small-sized phalanges [3]. The 1.0 mm or 1.2 mm K-wire holes can be used as drill holes for 1.2 mm or 1.5 mm sized small screws respectively. Thus, surgeons can choose ideal trajectories for screw insertion from the K-wire holes and replace the K-wire with small screws with or without stab incision. Third, surgeons might not consider the size and direction of the medullary canal; thus, the NMSS technique can be used in DIPJ and thumb IPJ arthrodesis with an extremely small medullary canal or in revision arthrodesis with a damaged medullary canal.

Our study has several limitations. First, the biomechanical results in this study could not prove that the NMSS technique is more stable than the AHCS technique. Compression force and mechanical stability differ between the types of HCS [25, 26], different small screw insertion techniques might have variable biomechanical stability, and our study did not include compression force in the NMSS technique. Our biomechanical results only showed that the stability of NMSS was comparable to that of AHCS in bending and torsional tests and suggested a theoretical basis for the NMSS technique. Second, in vivo biomechanical testing has limitations because of the absence of soft tissue, such as tendons and ligaments, and their effect on the rigidity of the constructs. Third, our clinical results used various fixation techniques such as using 1.2 mm screws, four screws, all unicortical screws, or all bicortical screws. Although union was achieved in all cases, the various fixation techniques had different biomechanical stability from the NMSS technique used in the biomechanical test. Thus, future studies should include measuring the compression force in the NMSS technique, a cadaveric biomechanical study, and a biomechanical comparison of various small screw techniques.

## Conclusions

The NMSS technique showed biomechanical stability comparable to that of the AHCS technique in DIPJ and thumb IPJ arthrodesis. Clinically, 15 consecutive DIPJ and thumb IPJ arthrodesis, including small-sized phalanx and flexed target positions, achieved union without severe complications using the NMSS technique. Thus, the NMSS technique could be used as a feasible option in DIPJ and thumb IPJ arthrodesis, especially when a small finger is indicated and a significant flexion angle is required.

## Supplementary Information

**Additional file 1:** Clinical result of the AHCS patient

## Data Availability

All data generated or analyzed during this study are included in this published article and its supplementary information files.

## Abbreviations

DIPJ: Distal interphalangeal joint
IPJ: Interphalangeal joint
HCS: Headless compression screw
AHCS: Axial headless compression screw
PIPJ: Proximal interphalangeal joint
NMSS: Nonaxial multiple small screws
SD: Standard deviation
VAS: Visual analog scale
DASH: Disabilities of the Arm, Shoulder, and Hand
