# Correction: Distal interphalangeal joint arthrodesis with nonaxial multiple small screws: a biomechanical analysis with axial headless compression screw and clinical result of 15 consecutive cases

**DOI:** 10.1186/s12891-023-06568-7

**Published:** 2023-05-30

**Authors:** Seung Hun Woo, Sang Ho Kwak, Hyo Seok Jang, Dong Hee Kim, Jang Hyeon Seo, Sang Hyun Lee

**Affiliations:** 1 Department of Orthopaedic Surgery, Pusan National University Yangsan Hospital, Yangsan, Republic of Korea; 2 Department of Orthopaedic Surgery, Inje University Haeundae Paik Hospital, Busan, Republic of Korea; 3 Department of Orthopaedic Surgery, Samsung Changwon Hospital, Sungkyunkwan University School of Medicine, 58, Paryong‑ro, Masanhoewon‑gu, Changwon‑si, 513‑53 Republic of Korea; 4 Jeil Medical Corporation, Digital‑ro 34, Seoul, Republic of Korea; 5 Department of Orthopaedic Surgery, Medical Research Institute, Pusan National University Hospital, Pusan National University School of Medicine, 179, Gudeok-ro, Seo-gu, Pusan, Korea, 602-739 Busan, Republic of Korea

**Correction:** ***BMC Musculoskelet Disord*** **23, 504 (2022)**

10.1186/s12891-022-05473-9

Following publication of the original article [1], the authors would like to add “Pusan National University School of Medicine” to Sang Hyun Lee’s current affiliation, so the affiliation would read “Department of Orthopaedic Surgery, Medical Research Institute, Pusan National University Hospital, Pusan National University School of Medicine, 179, Gudeok-ro, Seo-gu, Pusan, Korea, 602–739, Busan, Republic of Korea”.

The affiliation has been updated above and the original article [1] has been corrected.
