# Supplementary material for: Distal interphalangeal joint arthrodesis with nonaxial multiple small screws: a biomechanical analysis with axial headless compression screw and clinical result of 15 consecutive cases
Source: BMC Musculoskelet Disord. 2022 May 27;23:504. doi: 10.1186/s12891-022-05473-9 (PMC9137156; doi:10.1186/s12891-022-05473-9)
Supplement: Supplementary file 1 — Additional file 1: Clinical result of the AHCS patient [file 12891_2022_5473_MOESM1_ESM.docx]

Additional File. Clinical result of the AHCS patient

| Patient | Age(yr)/  Sex (M/F) | Laterality (R/L) | Finger location | Screw type | Duration of operation (min) | DIPJ and thumb IPJ fusion angle  (angle°/target position) | Follow up confirming union (Months) | Complication | Quick DASH | |
| --- | --- | --- | --- | --- | --- | --- | --- | --- | --- | --- |
|  |  |  |  |  |  |  |  |  | Preoperative | Postoperative |
| 1 | 55/F | L | 4 | Synthes 2.4mm | 65 | 3/ext | 6 | - | 34.1 | 9.1 |
| 2 | 60/M | R | 1 | Acutrak mini | 55 | 5/ext | 6 | - | 56.8 | 27.3 |
| 3 | 27/M | R | 3 | Synthes 2.4mm | 71 | 1/ext | 3 | - | 31.8 | 18.2 |
| 4 | 56/F | L | 2 | Synthes 2.4mm | 68 | -3/ext | 6 | Screw removal | 45.5 | 29.0 |
| 5 | 71/F | R | 3 | Synthes 2.4mm | 65 | 5/ext | 3 | - | 38.6 | 18.2 |
| 6 | 37/M | R | 1 | Acutrak mini | 58 | 3/ext | 3 | - | 61.4 | 29.5 |
| 7 | 65/F | L | 3 | Synthes 2.4mm | 67 | 6/ext | 6 | - | 56.8 | 13.6 |

M, male; F, female; R, right; L, left; DIPJ, distal interphalangeal joint; IPJ, interphalangeal joint; DASH, Disabilities of the Arm, Shoulder, and Hand
